# Hallmarks of stemness in mammalian tissues

**DOI:** 10.1016/j.stem.2023.12.006

**Published:** 2024-01-04

**Authors:** Joep Beumer, Hans Clevers

**Affiliations:** 1 Institute of Human Biology (IHB), Roche Pharma Research and Early Development, Basel, Switzerland

**Keywords:** adult stem cells, hallmarks, regeneration, plasticity, lineage tracing, niche, longevity, organoids

## Abstract

All adult tissues experience wear and tear. Most tissues can compensate for cell loss through the activity of resident stem cells. Although the cellular maintenance strategies vary greatly between different adult (read: postnatal) tissues, the function of stem cells is best defined by their capacity to replace lost tissue through division. We discuss a set of six complementary hallmarks that are key enabling features of this basic function. These include longevity and self-renewal, multipotency, transplantability, plasticity, dependence on niche signals, and maintenance of genome integrity. We discuss these hallmarks in the context of some of the best-understood adult stem cell niches.

## Teaser

Stem cell function is defined by their capacity to replace lost tissue through division. Beumer and Clevers put forth a set of six complementary hallmarks that are key enabling features of this function: longevity and self-renewal, multipotency, transplantability, plasticity, dependence on niche signals, and maintenance of genome integrity.

Every tissue is exposed to insults that require cellular turnover through the activity of adult stem cells (ASCs). In recent years, it has become clear that strategies to support the maintenance of homeostasis and repair after damage differ widely across tissues. For decades, the hematopoietic system has represented the textbook example of stem cell biology. Other tissues were anticipated to follow similar design principles: a hard-wired hierarchy with a rare, infrequently dividing (quiescent) stem cell at its base, which generates its progeny through asymmetric divisions and unidirectional differentiation pathways. However, the integrity of individual tissues and organs is subject to very different challenges based on size, function, internal architecture, and exposure to external insults. For instance, can dead cells simply be shed into the external milieu (skin, gut, lung, urinary tract) or do they have to be actively removed, e.g., by macrophages? Unsurprisingly, the translatability of the design principles of the blood-forming system has turned out to be rather limited. Moreover, considerable differences appear to exist between the same tissues across species.

**Figure 1:**
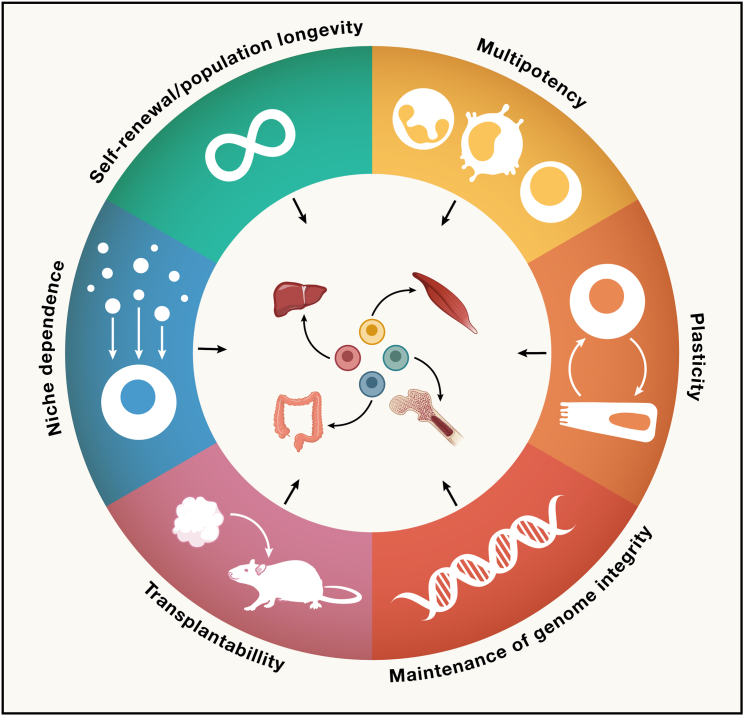
Hallmarks of stemness in mammalian tissues We describe a set of six complementary hallmarks that can be observed among tissue resident stem cells. These collectively enable the stem cell’s function to replace lost cells in the corresponding tissues.

We previously proposed a generalizable, functional definition of ASCs: the ability to replace lost tissue through cell division.^1^^,^^2^ Here, we elaborate on this topic and describe a set of six connected hallmarks that together describe the essential biological features of the various tissue stem cell mechanisms (Figure 1). Although these hallmarks cover distinct aspects of ASCs, they are complementary and—only when combined—can build a long-term (LT) tissue maintenance strategy. Through the lens of these hallmarks, we aim to take the reader through different maintenance strategies that have evolved in adult tissues.

## Self-renewal/population longevity

Arguably the most important trait of a stem cell population is to persist over the lifetime of the animal. This requires a high level of genome protection (discussed as a separate hallmark) to prevent the accumulation of DNA damage, which would result either in the demise of the mutated stem cells or in their malignant transformation. Additionally, stem cell division and commitment to differentiation have to be balanced. This can be regulated at the individual stem cell level through “hard-wired” asymmetric cell divisions, as currently still proposed in most textbooks: a stem cell division creates one daughter cell and one new stem cell. Yet, it can also be controlled at the stem cell population level: stem cells in a niche simply divide, and the progeny competes for space. Although all progeny have stem cell potential, the ones that are (randomly) pushed out of the niche become daughter cells, whereas the “lucky” ones remain as stem cells. The decline of tissue regenerative capacity during aging is generally believed to be correlated with an age-related demise of stem cell function. In this section, we will discuss a variety of self-renewal strategies observed in different tissue stem cell systems.

### Maintaining individual stem cells or a population of potential stem cells

The simplest model to ensure maintenance of a stem cell population over a lifetime is through invariant asymmetric cell divisions, regulated through cell-intrinsic factors.^3^ Such asymmetric cell divisions are well described in development of model organisms, such as the *Drosophila* neuroblast. Guided by unequal partitioning of intrinsic cell fate determinants or access to spatially distributed (extrinsic) niche factors, each stem cell division generates two differently fated cells.

Hematopoietic stem cells (HSCs) are among the most extensively studied ASCs. The traditional model describes multipotent, quiescent HSCs residing at the top of a rigidly choreographed hierarchy, giving rise to quadrillions of blood cells. Some evidence suggests that HSCs employ fixed asymmetric divisions.^4^ Asymmetric inheritance of organelles associated with cellular degradative machinery (particularly lysosomes), potentially guided by heterotypic interactions with osteoblasts, has predicted divergent fates of daughter cells.^5^^,^^6^ A similar regenerative paradigm is followed by satellite cells, the skeletal muscle stem cells.^7^ Though quiescent throughout most of life, individual satellite cells have the capacity to regenerate entire muscle fibers upon transplantation, producing 10,000s of mature cells.^8^ Upon damage, satellite cells initiate asymmetric divisions, with daughter cells presumably losing access to the basal lamina and subsequently committing to differentiation.^9^^,^^10^

Asymmetrically dividing stem cell populations must be able to switch to symmetric stem cell expansion in cases when some stem cells are lost or when extensive regeneration is required.^11^^,^^12^ The above studies have mostly relied on measuring asymmetry of divisions in *in vitro* assays. More work is needed to assess the balance of symmetric and asymmetric division in unperturbed dynamics *in vivo* and to understand the mechanisms that control this switch. Recent long-term *ex vivo* expansion as well as *in vivo* tracing and single-cell RNA sequencing studies have suggested extensive heterogeneity in the potency of individual HSCs. These studies question the predominant presence of invariant asymmetric divisions in the stem cells of the bone marrow^13^^,^^14^ (discussed in the section “multipotency”).

**Figure 2:**
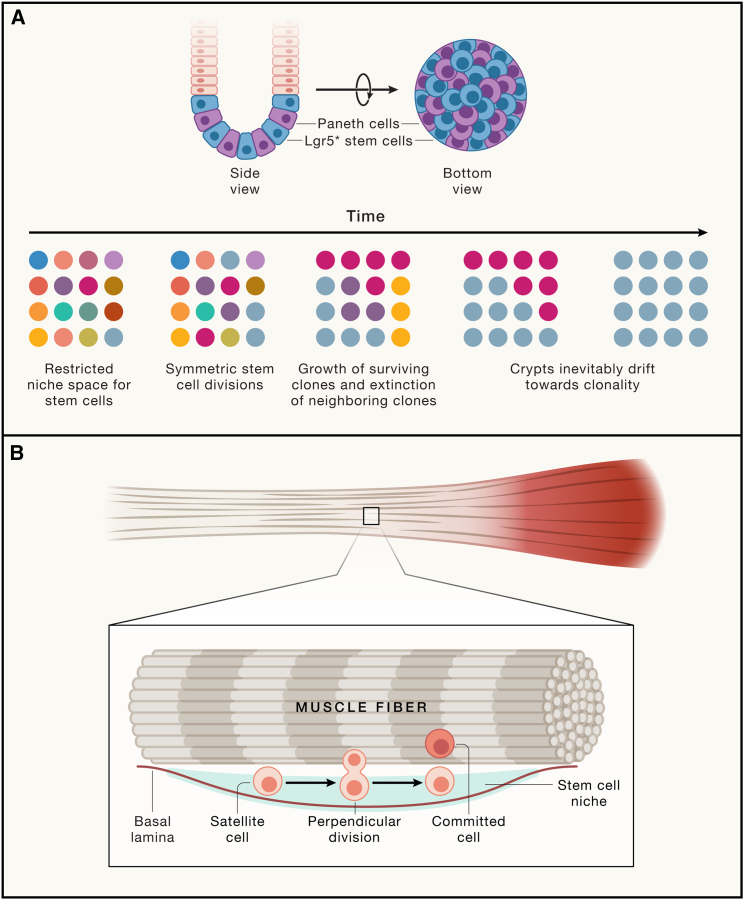
Maintaining individual stem cells or stem cell populations (A) Example of a “neutral drift” adult stem cell model. In the intestinal epithelium, stem cells residing at the crypt bottom divide symmetrically and compete for the limited niche space. Stem cell maintenance is guaranteed at the level of the population, but individual ASC clones are stochastically outcompeted while others gain dominance. (B) In the fibers of skeletal muscles, satellite cells can divide asymmetrically when the mitotic axis is perpendicular to the muscle fiber. The daughter cell retaining access to the basal lamina will maintain stem cell potential, whereas the other daughter cells commit to differentiation. In this example, the ASC displays asymmetry at the single-cell level, and individual clones are maintained.

It has gradually become evident that the majority of solid tissue stem cell populations with active homeostatic turnover appear to be maintained through symmetric divisions generating equipotent daughter cells (Figure 2). Depending on the position in their stem cell niche, these daughter cells will persist as stem cells or will enter a differentiation trajectory.^15^ This “neutral competition” of stem cells for niche space has now been described for the intestinal epithelium,^16^^,^^17^ stomach epithelium,^18^ esophageal epithelium,^19^ spermatogenesis in the testis,^20^ as well as for the epidermis and associated skin appendages.^21^^,^^22^^,^^23^^,^^24^ In these tissues, stem cell division leads to two potential stem cells that—depending on their access to stem cell niche factors—remain as stem cells or commit to differentiation. Rather than individual stem cells persisting long-term, the niche size imposes space constraints, thus strictly controlling the number of potential stem cells. This mode of competition is described as a neutral drift, which eventually leads to clonality: within a given niche, a tissue constantly yet stochastically loses stem cell clones, whereas other stem cell clones expand and eventually become dominant. Therefore, longevity cannot be determined at the level of the individual stem cell but should be ascribed to a stem cell population.

The neutral competition model suggests complete stochasticity for every stem cell division. However, both intrinsic and extrinsic factors such as the starting position in a niche or the acquisition of oncogenic mutations will affect the outcome of daughter cell fate. As a case in point, intestinal stem cells residing at the periphery of the stem cell niche have a higher likelihood to be displaced by dividing neighbors than those sitting at the bottom of the crypt.^25^ Although these cells are intrinsically equipotent (and in isolation would expand with equal efficiency), their position in the niche affects their competitiveness in the native tissue context. Committed cells that have exited the niche at the crypt bottom in some cases display retrograde movement and claim vacant positions in the niche space, reversing their trajectory back to a multipotent state.^26^ Oncogenic mutations affect the likelihood of maintaining longevity by gaining niche independence or even inhibiting healthy stem cells, essentially switching neutral competition to biased drift by increasing the odds of outcompeting neighboring stem cells.^27^^,^^28^^,^^29^

### ASCs can display quiescence or continious cell cycle activity

The traditional picture of a stem cell includes quiescence as one of its hallmarks, as this would prevent their exhaustion, minimize replication-associated DNA mutations, and thus ensure longevity. Quiescence is defined as a prolonged yet reversible cell-cycle exit. It is indeed tempting to speculate that minimizing stem cell division to an absolute minimum would allow for a reduced accumulation of DNA damage, preventing stem cell loss (or cancer). In this scenario, cell number expansion is overwhelmingly accomplished by rapidly dividing daughter cells. Although some adult tissues contain a quiescent stem cell population,^30^ this is not universal to all tissues. Importantly, quiescence is a relative concept rather than an absolute measure, as also quiescent stem cells inevitably undergo mitosis.

Quiescent cells are traditionally identified through long-term DNA (or chromatin) label retention and are hence referred to as label-retaining cells (LRCs). Of note, the various protocols will not only label putative quiescent stem cells but will also mark any terminally differentiated cell that exited the cell-cycle just after labeling. The HSCs and satellite cells represent well-described quiescent stem cell populations. The exact cell-cycle time of these populations has been difficult to determine due to slow incorporation of DNA labels as well as lack of definitive stem cell markers.^31^ Continuous bromodeoxyuridine (BrdU) supplementation to mice over 6 months revealed that 99% of the HSCs divide within that time frame.^32^ A shorter BrdU pulse of 10 days followed by a 70-day chase indicates that a significant fraction of HSCs (∼30%) are quiescent (dormant).^33^ Cells with the “deepest” quiescence are termed LT-HSCs and display the largest repopulation potential, as determined by competitive bone marrow transplantation.^33^^,^^34^ Yet, unperturbed hematopoiesis is predominantly driven by dividing cells (“short-term” [ST] HSCs) that are replenished by infrequent divisions from LT-HSCs.^35^ As ST-HSCs are long-lived and maintained largely independent of LT-HSCs, these can be viewed as a dividing population of stem cells. Depletion of the majority of LT-HSCs does indeed not perturb the generation of blood lineages during homeostasis, implying that ST-HSCs can sustain the bulk of steady-state hematopoiesis independently from LT-HSCs.^36^ Thus, these recent observations challenge whether the blood-forming system is predominantly dependent on dormant stem cells.

Stem cells in squamous epithelia such as skin, oral mucosa, and esophagus have long been thought to represent another quiescent population. The interfollicular epidermis (IFE) is organized in layers of gradually differentiating cells that eventually are shed. Administration of nucleotide analogs to neonatal mice revealed the presence of LRCs in the adult epidermis,^37^ and these LRCs were suggested to be stem cells based on their cellular positioning.^38^ This model proposes that these cells reside at the base of so-called epidermal proliferative units (EPUs), columns of the IFE emanating from single stem cells.^39^ In line with these early observations, lineage tracing based on genetic markers similarly indicated the presence of basal quiescent stem cells that asymmetrically divide 4–6 times per year to generate progenitor cells with a much higher turnover rate.^40^^,^^41^

Although lineage tracing allows for the analysis of the average behavior of proliferating cells over time, a live-cell imaging study has more accurately resolved single-cell fate decisions. Clonal dynamics across generations of cell divisions were assessed by making optical sections of identical parts of the skin in the same mice. Using different genetic tracers, this study did not confirm the presence of either asymmetric divisions or quiescent cells but rather indicated that equipotent basal cells constantly cycle with equal kinetics. The dynamic basal layer gives rise to stable units of differentiating cells—with little lateral exchange—and were re-termed epidermal differentiating units (EDUs).^24^ These findings were independently confirmed in a study that fitted cell division kinetics based on histone 2B (H2B)-dilution to published quantitative lineage tracing data.^42^ The contradicting observations can potentially be reconciled by the fact that different anatomical regions were assessed: quiescent IFE cells appear to be constrained to a specific part of the murine skin (interscale tail skin), whereas the majority of the murine skin lacks these.^21^^,^^40^^,^^41^^,^^43^ Finally, proximity to hair follicles is associated with higher turnover rate of basal stem cells,^44^ potentially related to factors secreted by growing hair.

The intestinal epithelium represents another tissue where a quiescent and dividing stem cell population have been proposed to coexist. Cell division is restricted to the crypt regions, invaginations in the epithelium that provide a safe space from the hostile gut lumen. A cycling stem cell population was proposed by Cheng and Leblond, who observed slender cells that divide and are interspersed between the larger post-mitotic Paneth cells.^45^ These cells were coined “crypt base columnar” (CBC) cells. Treatment with 3H-thymidine killed some CBCs, which were subsequently phagocytosed by neighboring CBCs. Isotope-labeled phagosomes could later be observed in all mature lineages, indicating their multipotency. More refined strategies using chemical mutagenesis tracing found further evidence for longevity of the CBC population, qualifying these cells as bona fide stem cells. Long-term “ribbons” of clones emanated from crypt bottoms to villus tips and included at least a single CBC.^46^^,^^47^ These findings were definitively confirmed using genetic lineage tracing based on the Wnt target gene *Lgr5*.^48^ The identification of Lgr5 as a specific marker allowed in-depth profiling, including their high cell division rate,^49^^,^^50^ neutral competition kinetics^16^^,^^17^ and *in vitro* expansion as organoid cultures.^51^

The alternative intestinal stem cell model describes a population at the so-called “+4-position,” 4 cell positions above the base of the crypt.^52^^,^^53^ These cells are reported as being sensitive to irradiation and retain DNA labels. Contrary to common belief, +4 cells were originally described as constantly dividing stem cells, whereas the mechanism behind their DNA label retention was proposed to result from asymmetric inheritance of newly synthesized DNA strands during replication. Known as the “immortal-strand hypothesis,” this mechanism has been proposed to allow putative stem cells to retain the parental template copies while handing over the newly synthesized DNA strands to their daughter cells, thus reducing accumulation of DNA damage in the stem cells. These findings were later refuted with more refined tracing strategies.^49^^,^^50^^,^^54^

In parallel to the discovery of Lgr5 as the CBC marker, multiple studies in mice proposed markers (Bmi1, mTert, Hopx, and Lrig1) for +4 cells and qualified these as stem cells based on genetic lineage tracing.^55^^,^^56^^,^^57^^,^^58^ All of these markers were, however, confounded by broad expression patterns based on extensive *in situ* hybridization profiling,^59^^,^^60^ as well as the first single-cell RNA sequencing studies that appeared around the same time.^61^^,^^62^

**Figure 3:**
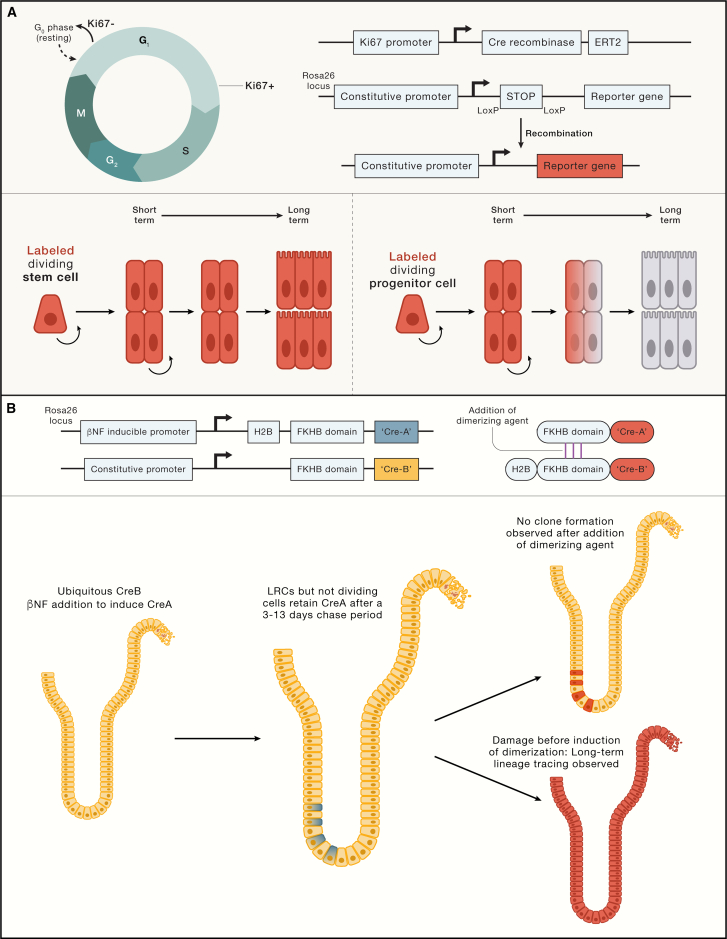
Determining stem cell potential in dividing and quiescent cells (A) Proliferating cells can be traced exploiting expression of KI67. KI67 is restricted to all cell-cycle phases except G0. A KI67-expression controlled Cre-recombinase can be used to trace dividing cells to assess their long-term clonogenicity and multipotency. When reporter recombination is initiated in a dividing stem cell (labeled in red, first scenario), long-term tracing will be observed. When the dividing cell is a committed progenitor, labeling is transient and will eventually be lost (second scenario). (B) Strategy to perform lineage tracing from slowly dividing or post-mitotic label-retaining cells (LRCs) in the murine small intestine. Cre-recombinase is expressed as two parts: the ubiquitously produced C-terminal CreB (in yellow) and the N-terminal CreA that is fused to histone 2B (H2B) (in blue). CreA expression can be induced by the addition of β-naphthoflavone (βNF), followed by cell-cycle-dependent dilution of the H2B-CreA protein. Both Cre-halves are additionally fused to an FKHB domain, of which fusion can be induced by a small molecule in order to reconstitute a functional enzyme and induce lineage tracing (traced cells will be red). CreB is constitutively expressed (yellow cells, left). Induction of CreA expression by βNF followed by a 3–13 days dilution will lead to a scenario in which only slowly dividing LRCs and post-mitotic cells retain both parts of the enzyme (blue and yellow cells, middle). The induction of dimerization does not yield long-term clone formation, suggesting that slowly dividing cells are not homeostatic stem cells (red cells, top). When epithelial damage is induced before dimerization of the Cre-domains, long-term tracing can be observed (bottom). These experiments evidence that LRCs are not homeostatic stem cells, but regain stemness upon damage.

Different molecular labeling strategies allow following the fate of actively dividing or quiescent cells, enabling the identification of putative stem cells with these functional characteristics (Figure 3). For example, the identity and fate of the enigmatic quiescent +4 cells were resolved using an innovative strategy of lineage tracing based on their slow cell-cycle kinetics. Rather than being multipotent, long-lived cells, +4 cells were found to represent a transient daughter cell state committed to secretory differentiation.^63^ A subsequent study identified *Mex3a* as a marker of cells that displayed features of CBCs (clonogenicity and multipotency) but also displayed slow cell division cycling kinetics.^64^ These cells were enriched at +3/+4 but could also be found at the bottom of the crypt. The discrepancy between the two studies could potentially be explained by the broader expression profile of Mex3a: it appears that these cells overlap with the secretory-biased LRCs but also with multipotent CBCs. Comparison of Mex3a-high versus -low cells shows enrichment of markers associated with secretory cell differentiation, suggesting that their predominant composition resembles LRCs.^64^ Another study generated a mouse model with fluorescently labeled Ki67, a proliferation marker, and crossed these with an Lgr5-reporter model. Lgr5-low/Ki67-low cells (suggestive of first CBC daughters with slow cell-cycle kinetics) were highly enriched for secretory differentiation markers and showed a high resemblance with LRCs described before (Figure 3).^63^ Lgr5-high cells were indistinguishable based on their Ki67 status.^65^ Therefore, a picture emerges that intestinal stem cells that exit the cell cycle are subsequently biased toward secretory differentiation. Of note, non-dividing Lgr5-expressing cells can be artificially induced *in vitro*.^66^ Although these cells have a bias toward secretory differentiation, these cells do not differentiate automatically but only when proliferation is reactivated.^66^^,^^67^ Therefore, active displacement from the stem cell niche by dividing cells is required for their commitment. The secretory fate bias may be regulated through lineage transcription factors that are directly controlled by cell-cycle-dependent kinases, which in turn are controlled by cell-extrinsic mechanisms such as Notch signaling. Similar observations have also been made in embryonic stem cells.^68^

Solid organs with a high turnover rate thus appear to preferably employ symmetrically dividing stem cells that compete for a limited niche space. Although long considered a hallmark of stemness based on HSC studies, quiescence (or DNA label retention) is not a *conditio sine qua non* for stem cell longevity. Even between anatomical regions of the same tissue type, considerable differences occur, such as in the case of the IFE. Moreover, recent insights emphasize the importance of inter-species differences, potentially as evolutionary adaptations to the lifespan, body size, or environmental stressors specific to the animal. A tour de force study by Toshiro Sato and colleagues utilized orthotopic transplantation of human intestinal organoid models to the murine colon combined with single-cell transcriptomics and lineage tracing^69^: this work demonstrated that human colonic Lgr5+ stem cells are predominantly slowly cycling, in contrast to their murine counterparts.

## Multipotency

Multipotency is the second stem cell hallmark. Stem cell potential refers to the ability to generate one or more differentiated cell types, known as uni- and multipotency, respectively (or pluripotency in the case of embryonic stem cells and induced pluripotent stem cells [iPSCs]). Both unipotent and multipotent tissue resident stem cells have been described. Stem cell potential can be observed in unperturbed conditions (default or homeostatic potential) or in the context of damage, where plasticity unleashes a larger range of multipotency. Stem cell fate decisions are overwhelmingly controlled through transcriptional and epigenetic regulators in response to extracellular signals and involve controlled expression of cell-type-specific genes as well as repression of other cell fates.

Under homeostatic conditions, many tissues harbor a single homeostatic stem cell population that generates all component cell types, such as in the case of intestinal CBCs or satellite cells. A compelling exception is the murine hairy skin, which is a continuous epithelium with diverse stem cell niches: the IFE and its appendages (hair follicles, sebum-producing sebaceous glands, and sweat glands). Each of these functional zones of the skin has its own stem cell population. Yet, these cells can display multipotency beyond their own “domain” and functionally replace one another when the skin is damaged. This plasticity is further evidenced upon transplantation of individual hair follicles that contribute to other parts of the skin.^70^^,^^71^

The epithelial glands of the stomach corpus similarly appear to harbor independent stem cell populations. The glandular units are partitioned into four distinct regions: starting from the bottom, these are termed base, neck, isthmus, and pit.^72^ Digestive-enzyme secreting chief cells populate the base of the gland, and mucus-producing pit cells face the stomach lumen, whereas other mature lineages are more randomly dispersed over the different regions. Radiolabeling experiments as well as transgene mutation-based tracing have suggested that proliferating stem cells occupy the isthmus and contribute to all stomach lineages through upward and downward migration.^73^^,^^74^^,^^75^ BrdU pulse-chase experiments have revealed, however, that the majority of the gland appears to be fueled by a multipotent isthmus cell, whereas the base of the gland is maintained separately.^76^ These findings could not be confirmed using genetic lineage tracing due to lack of specific markers of isthmus stem cells, yet a recent study circumvented the need of genetic markers by employing a mouse model allowing marker-free lineage tracing: a “confetti” tracer was stochastically induced yielding single-cell-derived clones, individually expressing one of confetti’s four fluorescent proteins. This experiment confirmed the presence of rapidly dividing isthmus stem cells but also showed that chief cells at the base of the gland are maintained separately,^77^ as proposed previously.^78^

### Unbiased lineage recording to reconstruct cellular differentiation hierarchies

Biases toward specific lineages can exist within apparently homogeneous stem cell populations. Genetic marker-based lineage tracing experiments have revealed most of our insights in the potential of the various types of ASCs.^79^ Yet, this tracing approach measures the “average” behavior of ASCs and may miss lineage biases of individual stem cells. Stem cell priming in the crypt can already be observed within the Lgr5 population, based on single-cell RNA sequencing.^80^

Emerging technologies allow dissection of the heterogeneity of clonal output at the single stem cell level and are starting to overcome limitations of traditional marker-based purification and tracing. HSCs represent a population of stem cells that displays heterogeneity in stem cell potential. In the classical model of hematopoiesis, LT-HSCs are proposed to fuel a multi-stage hierarchy of cells undergoing progressive fate restrictions and concomitant differentiation. Such studies have employed marker-based purification of these intermediate states and subsequent culture or transplantation.^81^^,^^82^ In this model, the first branching point of adult HSCs involves separation of the lymphoid and myeloid lineages in the form of common myeloid and lymphoid progenitors (CMPs and CLPs). The hierarchical model was later challenged by multiple reports that indicated earlier branching of self-renewing megakaryocyte progenitors,^83^^,^^84^ as well as the identification of HSCs that could generate lymphoid cells but only some of the myeloid lineage cell types (excluding megakaryocytes and erythroid cells).^85^ Heterogeneity at the level of HSCs themselves has further complicated the classical model, with some HSCs displaying lineage bias to myeloid lineages.^86^ Single-cell RNA sequencing of distinct HSCs and early progenitors combined with clonal outgrowth experiments have reinforced this more granular pattern, evidenced by early lineage priming and the gradual commitment to mature lineages.^14^^,^^87^^,^^88^^,^^89^

Recent technological advances allow the tracing of unperturbed hematopoiesis *in vivo* through stably integrating DNA marks. Rodriguez-Fraticelli et al. employed clonal labeling using a randomly inserting transposon and followed its inheritance through fluorescence-activated cell sorting (FACS) purification of mature cell types. This work provided evidence that indeed some of the traditionally defined LT-HSCs differentiate directly toward megakaryocytes, independently of other lineages.^90^ Chromatin accessibility of specific lineage marker genes in progenitor cells can inform on their propensities toward individual lineage allocation.^91^ Single-cell chromatin accessibility assays in hematopoietic progenitors further exposed such lineage biases, suggestive of early lineage priming.^92^ Alternative strategies, including tracing somatic mutations or telomere length in adult lineages, have allowed insights in developmental hierarchies in the human hematopoietic system.^93^^,^^94^ Such approaches infer the self-renewal capacity and fate of HSCs retrospectively through the accumulation of mutations in descendants of an ancestral stem cell division. These studies have provided evidence for a rapid expansion of HSCs in childhood, fueled by symmetric divisions, after which the pool sizes plateaus in adulthood. Human hematopoiesis (similar to murine models) appears to involve earlier branching of some blood lineages, indicated by the limited overlap in the number of mutations in granulocytes and T cells.^93^

The introduction of single-cell RNA sequencing and marker-free clonal tracing has created a perspective shift in the HSC field.^90^^,^^95^^,^^96^ The originally highly hierarchical tree-like model of differentiation involving multi- to unipotent progenitors is being replaced by a “landscape” view following a more fluid differentiation hierarchy with heterogeneity and lineage biases already occurring at the HSC level.^97^ HSC populations may therefore be best defined at a functional level, representing their fate potential. The gene-regulatory networks underlying these gradual changes in progenitor potential remain to be fully explored, enabled by recent technological advances such as *in vivo* CRISPR screens or new HSC culture models.^98^^,^^99^

## Transplantability

### Transplantation as a measure of stem cell activity

Before the development of more refined developmental tools, the ability of cells to expand and differentiate after transplantation has been the cornerstone assay to prove existence of stem cells. In the 1960s, McCulloch and Till developed a surrogate assay for stem cell potential (“spleen colony-forming unit”) by measuring whether engrafted bone marrow cells in the spleen could rescue lethally irradiated mice. They showed that the transplanted spleens developed clusters of cells derived from single engrafted HSCs and estimated the number of spleen colony-forming cells to be below 1 in 1,000.^100^ In the decades thereafter, transplantation assays have been developed for nearly all ASC populations. For example, single stem cells can form functional mammary outgrowths within 6 weeks when grafted in their native niche, the fat pad.^101^^,^^102^ We already highlighted the muscle skeletal stem cells, where single cells are capable of generating entire muscle fibers composed of 10,000s cells.^8^ The “transplantation” hallmark may, however, not be observed for every single ASC population and has limitations for quantifying clonogenicity of stem cells: the assay essentially surveys the additional ability of stem cells to survive the experimental handling when taken out of their native context, beyond its “true” stemness. A transplanted cell may further experience plasticity when grafted in the non-native environment (discussed in the next hallmark) and display stem cell behavior, even while not representing a homeostatic stem cell. The transplantation assay can therefore not be taken as an absolute measure to (dis)qualify cells as homeostatic ASCs.

### Cancer stem cells

Although beyond the scope of this review, it is worth mentioning that transplantability has been the key determinant of cancer stem cells. Pioneered by John Dick in the nineties for hematologic malignancies,^103^^,^^104^^,^^105^ the cancer stem cell-concept poses that tumors maintain a stem cell-like hierarchy resembling the stem cell hierarchy from their tissue of origin. Key experimental proof of “cancer stem cell-ness” has been the ability of cells sorted from to primary malignancy to re-establish the cancer upon xenotransplantation into immunodeficient mice.

### Stem cell therapy

After the nuclear bombing of Hiroshima and Nagasaki and the subsequent observation of the clinical consequences of radiation, experiments in irradiated mice indicated that transplanted stem cells could protect against the decline in blood lineages. The first allogeneic HSC transplantations were performed in the 1950s in patients that were irradiated or treated with chemotherapy yet yielded no clinical success. Therapeutic transplantations had to wait for another decade when the importance of the human leukocyte antigens (HLAs) and their matching was recognized. Edward Donall Thomas pioneered translation of HSC transplantation in acute leukemia in subsequent years, achieving clinical cures in a substantial number of cases for which he was awarded the Nobel Prize in 1990.^106^

The skin represents another area of clinical success. Howard Green and colleagues pioneered a culture strategy of human epidermal cells expanded on murine fibroblast feeder layers.^107^ Autologous epidermal stem cells taken from healthy, unaffected skin can be expanded and used to treat severe skin burns.^108^ Similar methods were applied to limbal stem cells of the cornea that could effectively cure blindness caused by alkali burns.^109^^,^^110^ Combination of Howard Greens’ culture system with gene therapy rescued a 7-year-old child with a lethal blistering disease, caused by a laminin mutation: about 1 m^2^ of skin was generated by culturing autologous epidermal stem cells, retrovirally transduced with the wild-type laminin gene. The resulting cellular sheet was transplanted and yielded a nearly normal skin, resistant to mechanical stress and devoid of blisters.^111^^,^^112^

Combined developments in CRISPR-based genetic engineering and novel organoid models hold promise for stem cell therapy. For example, organoid technology now enables the expansion of salivary gland stem cells *in vitro*. Transplantation of these organoids has effectively restored saliva production in irradiated mice.^113^ Loss of saliva production is a serious complication in cancer patients irradiated in the head region, and the first clinical study using autologous organoids has recently been initiated in the Netherlands. Similar trials are currently underway for the treatment of ulcerative colitis, where transplanted intestinal organoids may restore some of the epithelial regenerative abilities. Heart failure represents another indication that could greatly benefit from stem cell therapy, yet many clinical trials have previously failed based on misidentified adult cardiac stem cells. More encouragingly, recent strategies to derive cardiomyocytes from iPSCs have led to trials in patients with severe heart failure. Pre-clinical experiments in primates have evidenced that these cells can indeed regenerate heart tissue, displaying electric coupling to host cardiomyocytes.^114^ The first patients have recently been treated with a direct injection of these cardiomyocytes into the heart muscle during a coronary bypass.

## Plasticity

**Figure 4:**
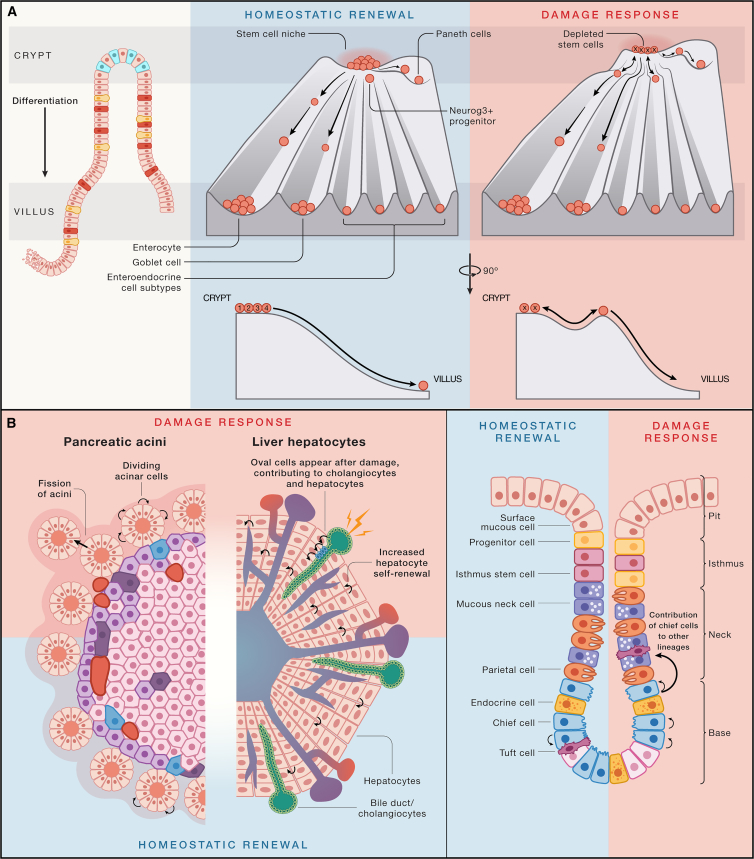
Plasticity in tissues with fast and slow turnover (A) Waddington landscape of the intestinal crypt in homeostasis (left) and upon damage to the stem cell compartment (right). In homeostasis, cells gradually commit and differentiate while leaving the stem cell zone and losing access to stem cell niche factors. Yet, during damage and loss of stem cells, the majority of crypt cells can readily reverse their commitment due to permissive chromatin when exposed to stem cell signals. Stem cells at the “edge” of the stem cell niche (numbers indicate the cell position counting from the bottom of the crypt) are more likely to lose access to key stem cell signals and differentiate. (B) Examples of tissues that employ self-replicating mature cells to sustain tissue renewal. In the damaged pancreatic acini, acinar cells can increase self-renewal and induce an accelerated rate of acini fission. Damage to the liver causes the emergence of oval cells from cholangiocytes that can both generate both new cholangiocytes as well as hepatocytes. Chief cells at the bottom of the stomach glands can self-replicate during homeostasis to sustain their pool, but damage endows these cells with expanded lineage potential.

Conrad Waddington postulated the iconic “Waddington’s landscape” in 1957, a theoretical framework to describe collections of cells progressively becoming lineage-restricted during development.^115^ The downward flow and increase in valley depth are metaphorical representations of the increasing energy barriers for return toward pluripotency (“dedifferentiation”) and cell state transition (“transdifferentiation”).^116^ It is worth noting that even though embryogenesis occurs in a physically protected environment, Waddington already envisioned the landscape to allow for a level of cellular plasticity to compensate for developmental challenges. Waddington’s landscape provides a framework for embryonic development, but adult tissues essentially adhere to the same guiding principles. The increased exposure to environmental perturbations interfering with homeostatic renewal does require more extensive plasticity. Permissive epigenetic wiring enables adult cells to freely switch their cell state in response to damage signals. Small-molecule perturbations in organoids combined with single-cell RNA readouts now enable to define how cell potential dynamically changes in response to such signals.^117^ Waddington landscapes can be drawn for every adult tissue in the context of homeostasis, yet they will most likely change in the context of damage: damage signals are effectively lowering the barriers to fate transitions (Figure 4). They also may change the “slope” of the landscape such that differentiating cells can fully revert to a multipotent ASC state. Plasticity can be viewed as the third defining hallmark of ASCs (and their progeny). In this section, we illustrate these “injury-response” dynamic landscapes in the context of rapidly renewing tissue (gastrointestinal tract epithelium), as well as a tissue with limited adult turnover (liver).

### Gastrointestinal tract

The small intestinal epithelium displays the fastest turnover of all adult solid tissues, renewing approximately every 5–7 days under physiological conditions. In homeostasis, CBCs generate the five main epithelial lineages: the absorptive enterocytes and the secretory lineages that include mucus-producing goblet cells, hormone-secreting enteroendocrine cells (further subdivided into five subsets), Paneth cells that produce the stem cell niche factors, and immunomodulatory Tuft cells.^118^ Regenerative responses in the epithelium were long believed to require the “reserve” quiescent stem cell population at the +4 position (discussed above). CBCs can be ablated using irradiation, chemotherapy, or by genetic approaches such as Lgr5-driven diphtheria toxin receptor expression.^119^^,^^120^^,^^121^^,^^122^ Subsequent regeneration involves the generation of new Lgr5+ CBCs from multiple non-CBC sources.^122^ Genetic lineage tracing performed in damage models has revealed that essentially all committed progenitor cells can revert to a stem cell population to subsequently contribute to tissue renewal, including LRCs.^63^^,^^123^^,^^124^ Thus, the return of these cells to the stem cell niche at the bottom of the crypt, combined with potential damage signals transduced through Hippo/YAP1,^118^^,^^125^ is sufficient to stimulate their dedifferentiation. Permissive chromatin states allow progenitors to smoothly “undo” their lineage commitment.^126^ It remains to be determined at what point the epigenetic resistance to this state reversion becomes irreversible. Enteroendocrine cells have been proposed to retain damage-evoked stem cell potential, but it is unclear if this truly extends to fully differentiated cells, or only to committed progenitor cells.^127^ Mature Paneth cells were similarly suggested to reacquire stem cell potential after damage, and these cells may be favored by residing in the stem cell niche at the crypt base.^128^^,^^129^ Nonetheless, non-mitotic differentiated cells certainly display low outgrowth efficiency when cultured in the presence of the key growth factors mimicking the stem cell niche.^65^

Other parts of the gastrointestinal tract exhibit similar levels of plasticity, such as dedifferentiation of secretory progenitors in the colon.^130^^,^^131^^,^^132^ The stomach represents a more curious case, where differentiated cells can contribute to regeneration by increased cell-cycle activity. Upon damage, chief cells residing at the base re-enter the cell cycle and initiate the expression of mucous cell markers, resembling a metaplastic response. This state, known as spasmolytic polypeptide-expressing metaplasia (SPEM), allows for rapid regeneration of the damaged epithelium but can also represent a precursor of gastric cancer.^78^^,^^133^

### Liver hepatocytes

The extensive capacity of the liver to regenerate is beautifully symbolized by the Greek myth of Prometheus, who was punished by Zeus and had to endure eternal daytime feasting of an eagle on his liver, followed by tissue regeneration during the night. Although the liver does not appear to contain professional stem cells, it is arguably the most regenerative organ of the mammalian body. How does this work? The liver is essentially composed of two key endodermal cell types: the cholangiocytes (or bile duct cells) and the hepatocytes. Both these cell types display a very-low turnover rate during homeostasis.^134^ Surgical removal of part of the liver, known as hemi-hepatectomy, induces rapid proliferation of mature hepatocytes and allows the organ to regain its previous size, without overt dedifferentiation.^135^ Up to 90% of murine and 40% of human hepatocytes are polyploid, yet this state does not appear to interfere with their proliferative capacity.^136^ Axin2-positive diploid hepatocytes residing in a niche of high Wnt signals around the central vein have been proposed to be endowed with the highest regenerative capacities, fueling renewal of polyploid hepatocytes.^137^ More recent studies using random clonal labeling have indicated a more uniform regenerative capacity across liver hepatocytes without zonal dominance.^138^^,^^139^ The extent of the regenerative capacity of mature, functional hepatocytes is further emphasized by their long-term expansion as 3D *in vitro* organoids.^140^^,^^141^

Chronic injury to hepatocytes by viral infection or toxins induces a different regenerative response, characterized by the appearance of so-called oval cells.^142^^,^^143^ These cells are believed to derive by rapid dedifferentiation of cholangiocytes into a bipotent proliferative state, capable of generating both new cholangiocytes as well as hepatocytes. This transition is captured in cholangiocyte-derived organoids^144^ and involves rapid epigenetic reprogramming.^145^ The formation of mature hepatocytes from mature cholangiocytes *in vivo* has been evidenced by a genetic model of blocked hepatocyte proliferation.^146^^,^^147^^,^^148^

Thus, low-turnover tissues such as the liver can bestow proliferative stem cell-like capacities onto hepatocytes or cholangiocytes, whereas tissues with higher turnover such as the gastrointestinal tract epithelia may exploit reacquisition of multipotency of committed cells when the actual stem cells are lost. Two other well-established examples where fully differentiated cells act as stem cells are the alveolar type II (ATII) cells of the lung alveoli^149^^,^^150^ and the acinar cells of the pancreas, which can self-replicate upon damage to the organ^151^^,^^152^ (Figure 4). Future work is needed to systematically determine the “point of no return” of cells along their differentiation trajectory. The hepatocytes and ATII cells may represent one extreme where fully differentiated cells can “double” as stem cells. The hematopoietic system represents the other extreme, where efficient reprogramming of committed blood lineages to an HSC state requires the overexpression of eight different transcription factors (double the number of the “Yamanaka factors”), illustrating the magnitude of this barrier.^153^ Thus, the hematopoietic system appears to be the exception rather than the rule, possibly because—as a diffuse, liquid “organ”—cells that exit from the HSC niche are immediately deprived of local morphogen signals that in solid tissues aid in “remembering” cell fate.

## Niche dependence

In 1978, Ray Schofield proposed the concept of a stem cell niche to explain the maintenance of multipotency versus lineage commitment of HSCs.^15^^,^^154^ This physical space contains the signals that maintain stem cell features. The stem cell niche constitutes the pinnacle of Waddington landscape from which the maturing cells roll downhill. The niche provides the physical guardrails within which stem cells can neutrally compete to retain their identity, and the size of the niche thus controls the numbers of stem cells. Niches can come in many forms and shapes. In the gut epithelium, each of the billions of crypts contains a tiny stem cell niche at its base; in the epidermis, the basal layer forms a single very large 2D structure that wraps around the entire body. Schofield already noted that a return of a cell to the stem cell niche may drive its dedifferentiation. Acquisition of niche independence is one of the features of malignant transformation, allowing cells to display stem cell hallmarks outside their natural limits. In this section, we will discuss different technological approaches to defining stem cell niches and highlight how these have synthesized an in-depth understanding of the molecular control of intestinal stem cells.

### Defining the stem cell niche

An important quest for stem cell biologists is the identification of the niche factors that balance self-renewal and lineage commitment, as well as defining the cellular sources of these signals. Niche signals include soluble factors, cell-bound molecules, and extracellular matrix components. Due to the complexity of niches, our current knowledge remains rather rudimentary. Most insights have been obtained from transgenic mouse models in which individual signals are genetically manipulated. Traditional immunohistochemistry and *in situ* hybridization are limited in sensitivity and throughput in terms of mapping the sources and targets of signaling interactions. Recent developments in single-cell genomics have created a wealth of additional hypotheses of components constituting unique niches. Single-cell RNA sequencing can be used to produce comprehensive maps of putative ligand-receptor interactions in niches.^155^^,^^156^ Such efforts are now allowing to optimize culture conditions of different *in vitro* models. In the past years, developments in spatial transcriptomics have started to provide information on physical distances between potential sender and receiver interactions.^157^ Alternatively, highly efficient isolation of specific groups of cells combined with single-cell RNA sequencing can yield similar insights.^158^ Innovative mouse models furthermore can label cells that are in close proximity of each other, evidencing functional cell-cell interactions in real time.^159^^,^^160^ Such approaches can now be used to purify stem cells and nearby niche sources at scale.^161^ Although these experiments only indicate what kinds of close proximity interactions exist, they may in the future be functionally manipulated using specific cell-cell interaction-activated gene editing modules.^162^

Another approach to characterize stem cell niches is through reconstructing these with soluble factors and matrix components *in vitro*.^163^ The first solid tissue mammalian cell culture was established for the human epidermis by expanding keratinocytes on a mouse fibroblast feeder layer.^107^ The subsequent decades of stem cell research have led to the development of organotypic cultures for the majority of adult epithelia. These can now be expanded in an extracellular matrix-mimicking gel (Matrigel) enriched with growth factors mimicking the stem cell niche.^164^ Growth factor screens have enabled the dissection of minimal stem cell niches, as well as the morphogens required for differentiation into different lineages.^164^ Designer matrices are informing on the requirement of different extracellular matrix composition and stiffness to maintain stemness or promote differentiation.^165^^,^^166^^,^^167^

The intestinal crypt is arguably the best-defined stem cell niche. Wnt factors, first identified as stem cell drivers in the gut,^168^ are redundantly produced by myoepithelial cells that surround the crypt base^169^^,^^170^ (Wnt2b) as well as by the epithelial Paneth cells,^171^ themselves daughters of the stem cells that they support. Paneth cells also provide Notch signals to block the default differentiation of their neighboring stem cells into the secretory lineage.^172^^,^^173^ Additional signals that emanate from the stroma around the crypt base are the Wnt-amplifying R-spondins (ligands of the Lgr5 stem cell marker) and BMP inhibitors such as the Gremlins, which also block differentiation.^118^^,^^157^ Stem and progenitor cell proliferation is driven by epidermal growth factor (EGF).^66^ Daughter cells that move away from the crypt base toward the villus tip experience decreasing Wnt signals while moving up a BMP gradient established by the villus stroma.^174^^,^^175^ During this process, stochastic Notch signals are key drivers of the diversification of cell fates.^172^ Murine small intestinal organoids can indeed be expanded in a very limited niche consisting of the Wnt amplifier R-spondin, the BMP inhibitor Noggin, and EGF, while suspended in Matrigel.^51^ Organoid culture conditions have now been derived for the majority of human epithelial stem cell populations. As a general rule, epithelial stem cells depend on a minimum of three types of niche signals: (1) Wnt/Rspondin factors, (2) activators of tyrosine kinase receptors such as EGF, and (3) inhibitors of TGF-β/BMP signals. This uniformity extends to other vertebrates such as snake venom gland, which can be propagated *in vitro* as organoids using same growth factors as used for human systems.^176^ Thus, niche factors that maintain epithelial stem cells in adulthood appear to be shared across tissues and vertebrates.

Stem cell output can influence the niche as part of a feedback loop. Maturing keratinocytes can modulate cell division and differentiation of underlying basal stem cells through increased contractility.^177^ In the hair follicle, transient primed stem cells and quiescent stem cells coexist, each fueling parts of the hair growth cycle.^178^ Immediate daughter cells of primed stem cells produce sonic hedgehog, which in turn is essential to active quiescent stem cells. The latter can replenish primed stem cells, completing a complex niche circuitry that additionally integrates non-epithelial signals from the dermal papilla.^178^

## Maintenance of genome integrity

Beyond a control in multipotency, Ray Schofield suggested the stem cell niche to play an important role in preventing accumulation of genetic mutations, for example, through limiting the cell division rate. As discussed above, not all ASC populations adhere to the hematopoietic model of quiescence, raising important questions as to how stem cells protect their genomic integrity to prevent malignant transformation or irreversible stem cell loss. The immortal-strand hypothesis was proposed as an alternative stem cell-specific mechanism, yet this model has been refuted by recent studies,^54^ with the exception of the muscle satellite cell.^179^ Taking into account that stem cell populations are long-lived, the chance that a harmful mutation may acquire clonal dominance should be minimized. In this section, we will discuss different safeguards ranging from cell-intrinsic to population-level mechanisms.

### ASCs limit accumulation of DNA damage

The assumption that stem cell division rate necessarily correlates with mutational accumulation has been challenged by multiple independent observations. HSCs critically depend on non-homologous end joining (NHEJ) for repairing genomic damage, as homology-dependent repair (HDR) requires a cell division to exploit the homologous sister chromatid.^180^ NHEJ is more error-prone than HDR, and indeed, HSCs may accumulate more genomic rearrangements that can contribute to transformation, compared with their dividing daughter cells.^180^ Whole-genome sequencing of clonal multipotent stem cell-derived organoids from primary liver, small intestine, and large intestine biopsies from donors aged 3–87 years has allowed the quantification of single base mutation rates in these three tissues.^181^ Although the liver essentially represents a non-dividing tissue and the gut epithelium is among the fastest cycling tissues, all 3 cell types accumulate approximately 40 mutations per stem cell per year in a linear fashion. This implies that quiescence or slow cell cycling in itself does not protect against mutations.^181^ Known as Peto’s paradox, larger animals tend to develop less cancer despite having more cells that could transform. A recent study compared the accumulation of DNA mutations across the intestines of different species.^182^ Although these species had a 30-fold lifespan- and 40,000-fold body mass variation, the mutational burden at the end of life only differed 3-fold.^182^ Thus, evolution appears to be able to endow stem cells with genome integrity capabilities that match the size and lifespan of the pertinent species.

Rather than tuning cell division rate, some stem cell populations appear to employ a more extensive DNA repair machinery than their committed daughter cells.^183^ Mammary gland stem cells were found to contain higher levels of NHEJ activity than their differentiated counterparts.^184^ Hair follicle stem cells express higher levels of anti-apoptotic proteins and display an efficient NHEJ-mediated DNA repair, preventing cell death while requiring a short pulse of p53 activation during DNA damage.^185^ CBCs in the gut display high expression of a large set of HDR genes, regulated by WNT signaling.^186^ Telomere shortening is prevented by high expression of telomerase in ASCs, such as the gut and liver.^49^^,^^187^ In addition, stem cells can prevent the accumulation of mutations through metabolic adaptations.^186^ HSCs for example predominantly display a low glycolysis metabolic activity, limiting reactive oxygen species (ROS) production.^188^ Mutation rates in the spermatogonia, the testis stem cells that create sperm cells throughout life, are over 20-fold lower than in other somatic stem cell types (∼2 mutations per year).^189^ Spermatogonial stem cells are unlike any other ASC in that all somatic mutations will be passed on to the next generation. Apparently, spermatogonia are uniquely endowed with mechanisms to keep their genomes “clean.” It will be of great interest to understand how these unusual stem cells accomplish this feat.

### Population dynamics can affect mutation fixation in ASC populations

Beyond cell-intrinsic strategies to maintain genomic integrity in individual stem cells, dynamics at the level of a stem cell population may help to prevent clonal fixation of cells with harmful mutations. As tissues that utilize neutral competition in their niches do not depend on maintaining individual stem cells, clearance of damaged stem cells could be one way by which this could be achieved.

The architecture of the stem cell niche may play a key role in maintenance of ASC genome integrity at the population level. As an example, the layout of the intestinal epithelial stem cells space may reflect a two-pronged strategy to avoid the fixation and spread of stem cells carrying an oncogenic mutation. First, only ∼15 stem cells reside in individual crypts, and most of these will disappear due to neutral drift even if they carry mutations. Second, if a mutant stem cell successfully colonizes an entire crypt, it is essentially locked in a small space and cannot develop into an ever-growing clone to increase chances for second and third oncogenic hits. The epidermis or the bone marrow represent two examples at the other extreme: their stem cells essentially live in a single, very large space that spans the entire body; thus, the activities of a maverick stem cell will not be ringfenced. As a case in point, we mention chronic myeloid leukemia and paroxysmal nocturnal hemoglobinuria in which a single aberrant HSC can overtake the entire bone marrow space while continuing to produce cells of all blood lineages.^190^^,^^191^

Genotoxic or endoplasmic reticulum (ER) stress in HSCs can induce differentiation or cell death.^192^^,^^193^^,^^194^ The reverse can also occur: retrograde movement and dedifferentiation of committed cells in the gut have been proposed to protect against mutation fixation, as it effectively increases the number of wild-type stem cells to compete with mutant stem cells.^195^ It remains to be established if such events occur outside experimental settings. Harmful clonal dynamics have been described more extensively. Oncogenic mutations in the esophagus and small intestine create a competitive advantage for mutant stem cells, increasing the odds of clonal fixation.^196^^,^^197^^,^^198^ These clonal dynamics are obviously shaped by changes in niche sensitivity but also by potential immune responses. There is currently only a rudimentary understanding of how local immune responses may influence clonal dynamics in the earliest phases of carcinogenesis, but immune clearance may prevent damaged stem cells from reaching dominance. This has indeed been shown in the small intestine, where intraepithelial lymphocytes target adenomatous polyposis coli (APC)-mutant cells.^199^

### Concluding remarks

There appears to be no single ASC hierarchy design that fits into a generalizable mold: evolution has created a variety ASC designs. The evolutionary forces that drive these differences remain nebulous, but one could speculate that these include the extent of environmental and occupational damage sustained by individual tissues, the ease of cell disposal, and tissue tolerance to somatic mutations (spermatogonia being at the extreme minimum of such tolerance). Though stem cell hierarchies appear quite distinct between tissues, all models adhere to a set of features or “hallmarks” that we describe here. We believe that these ASC hallmarks provide a framework to understand stem cell behavior in postnatal life of vertebrates. Strictly, the stemness hallmarks should be viewed at the stem cell population level, as individual stem cells do not necessarily display longevity. Plasticity among non-homeostatic stem cells allows a return to a stem cell-like state and appears to be the rule rather than the exception.

Genetic marker-based lineage tracing has informed the field on many stem cell hierarchies but has been similarly at the center of intense debates when the marker genes were more broadly expressed than anticipated. Emerging technologies based on CRISPR “scarring” and barcoding now allow unbiased tracing of large numbers of clones over time and can be coupled to single-cell transcriptomic and epigenetic readouts. This information can be extracted to deduct complex lineage relationships and unbiasedly assess potency of progenitors and putative stem cells.^200^^,^^201^^,^^202^^,^^203^^,^^204^ Innovative modifications even allow the extraction of information around the order in which the edits have occurred. These true molecular recorders will enable more extensive dissection of lineage history including branching points over physiologically relevant time periods.^205^^,^^206^^,^^207^ Some of the approaches are readily applicable to human lineage reconstructions. Such work can be enriched with human organoid models to inform on cross-species differences in terms of stem cell renewal strategies. These types of comparisons may even yield new insights into relative frequencies of cancers.

Our understanding of stem cell niches remains relatively rudimentary, yet emerging technologies including spatial transcriptomics generate new insights on the signals that drive stem cell behavior. Organoids represent reductionist, self-organizing stem cell systems that allow the bottom-up reconstitution of stem cell niches. Functional studies of the niche are enabled by combining these tissue avatars with specific soluble growth factors, designer matrices, or co-cultures with specific cell populations. This is particularly powerful when supported by bioengineering approaches to control spatial organization.
